# Longitudinal Relationship Between Sitting Time on a Working Day and Vitality, Work Performance, Presenteeism, and Sickness Absence

**DOI:** 10.1097/JOM.0000000000000809

**Published:** 2016-08-08

**Authors:** Ingrid J.M. Hendriksen, Claire M. Bernaards, Wouter M.P. Steijn, Vincent H. Hildebrandt

**Affiliations:** Expertise Centre Lifestyle, Netherlands Organisation for Applied Scientific Research TNO, Leiden (Drs Hendriksen, Bernaards, Hildebrandt); Body@Work Research Center on Physical Activity, Work and Health TNO-VU/VUmc, Amsterdam (Drs Hendriksen, Bernaards, Hildebrandt); and Department of Urban Environment and Safety, Netherlands Organisation for Applied Scientific Research TNO, Leiden, The Netherlands (Dr Steijn).

## Abstract

**Objective:**

The aim of this study was to explore the longitudinal relationship between sitting time on a working day and vitality, work performance, presenteeism, and sickness absence.

**Methods:**

At the start and end of a five-month intervention program at the workplace, as well as 10 months after the intervention, sitting time and work-related outcomes were measured using a standardized self-administered questionnaire and company records. Generalized linear mixed models were used to estimate the longitudinal relationship between sitting time and work-related outcomes, and possible interaction effects over time.

**Results:**

A significant and sustainable decrease in sitting time on a working day was observed. Sitting less was significantly related to higher vitality scores, but this effect was marginal (*b* = −0.0006, *P* *=* 0.000).

**Conclusions:**

Our finding of significant though marginal associations between sitting time and important work-related outcomes justifies further research.

Sedentary behavior, defined as any waking behavior characterized by an energy expenditure of 1.5 METs (metabolic equivalents) or less while in a sitting or reclining posture,^1^ has emerged as a risk factor for premature death and several chronic diseases, independent of the amount of physical activity.^2–5^ In many jobs, the workplace is an important source of prolonged sitting: about one-third to half of our daily sitting time is work-related.^6–9^ One can expect that occupational sitting will increase even further the coming years by ongoing automation and use of IT. Research on the association between occupational sitting and major work-related outcomes such as vitality, work performance, presenteeism, and sickness absence is scarce. There are three cross-sectional studies exploring these relationships: one examined the association between sitting and vitality and work performance,^10^ and two studied the relation between sitting and presenteeism.^11,12^ Munir et al^10^ showed that those UK office-workers who reported good work vigor (ie, vitality) were less likely to report high occupational sitting times, and significant correlations were found for higher job performance with lower occupational sitting times. In Australian office employees, Brown et al^11^ reported significant associations for time spent in sedentary behavior before and after work and presenteeism, but not during work time. And in Australian workers who completed an online survey, Guertler et al^12^ unexpectedly found that lower work-related sitting time was significantly associated with higher presenteeism when controlling for all lifestyle behaviors.

Recently, several reviews have been published on the effects of workplace interventions to reduce sitting at work with a specific focus on active workstations, which also included work productivity as a secondary outcome measure.^13–16^ Although they showed that active workstations can decrease sitting time, no or conflicting effects on work performance were found. However, the main focus of these studies was to examine whether active workstations impaired work performance relative to the traditional seated condition. Besides, in some studies, work performance was not defined at all, while other studies showed a large variety in definitions of work performance (including “concentration,” “typing performance,” and “production levels”), which makes comparison very difficult. Finally, in the recent review of Neuhaus et al,^14^ the pooled effect size of the meta-analysis showed a significant decrease in sedentary time during the workday after implementation of active workstations, but no significant changes in presenteeism and absenteeism. In none of these reviews, the possible association between changes in sitting time and changes in work-related outcomes was explored.

Nevertheless, it is plausible that the adverse health effects of prolonged sitting can result in reduced employability of workers, due to a lower vitality and work performance and higher presenteeism and sickness absence. These adverse effects may constitute a great concern for employers, because they can result in reduced productivity. From that viewpoint, it seems highly relevant to pay attention to sedentary behavior when designing and implementing health promotion programs at the workplace. This study describes such a program, which was implemented at a large Dutch insurance company involving workers with mainly sedentary tasks. The aim of this study is to examine to what extent changes in sitting time during working days induced by a health promotion program are associated with changes in vitality, work performance, presenteeism, and sickness absence.

## METHODS

### Intervention

The intervention program targeted (self-) awareness and knowledge on vitality, lifestyle, and physical activity practices with the aim to increase self-management to perform healthy behaviors. The intervention consisted of a joint kick-off meeting, two vitality training sessions (both taking half a day) and two workshops (participants could choose out of four different topics) for all employees. In addition, a training and two intervision sessions were organized for supervisors. All employees were offered opportunities for individual coaching. Incentives included the distribution of water and fruit and permission to perform the activities during working hours. More information on the intervention program and its effectiveness is published elsewhere.^17^

With regard to sedentary behavior, the intervention was intended to create awareness on the number of hours participants were sitting on a working day. This was realized by communicating the individual results of the questions on sitting time with the participants online as well as during the health check by a vitality coach, during a workshop on effective physical activity, and during the training of the supervisors. However, reducing sitting time was not a primary focus of the intervention, rather increasing physical activity was emphasized.

The intervention lasted five months and was introduced stepwise in five diverse company departments between September 2012 and May 2014.

### Design and Study Population

The effectiveness of the intervention and the relation between sitting time and the outcome measures was evaluated by comparing outcome measures before and after the intervention (pre-post design, no control group). Measurements were done at baseline (T0), at the end of the intervention (T1), and 10 months after finishing the intervention (T2).

All employees of a division of a Dutch insurance company (mainly white collar workers, in total 502 employees including 52 supervisors) were invited to participate. Before the start of the program, all participants received information about the intervention and were asked to give written informed consent to retrieve information for research purposes from the intervention measurements and sickness absence data from company records.

### Measurements

Data on age, gender, education [included as 8-point scale from (0) no education to (7) bachelor or master degree], and employment status (working hours per week) were determined at baseline by a standardized self-administered questionnaire (the Energy & Performance Scan, EPS). Body weight and body height were collected during health checks that occurred at T0 and T1. Body mass index (BMI) was calculated by dividing the body weight (kg) by the square of body height (m^2^), and categorized as less than 25 kg/m^2^ (normal weight) and at least 25 kg/m^2^ (overweight). Participant's moderate to vigorous physical activity (MVPA) level was measured at all time points using the EPS as well. MVPA was assessed by questions on the usual number of hours and minutes per day of MVPA (eg, walking, cycling, and gardening) during each day of the week, while including only those activities that lasted at least 10 minutes.

Data on sitting time, vitality, work performance, and presenteeism were collected at all three time moments using the EPS. Sitting time was measured by asking how many hours and minutes were spent sitting on a regular working day during (1) work, (2) work breaks, (3) travelling to/from work, and (4) leisure time. Questions on vitality and work performance were derived from validated questionnaires and modified to fit the target group. Vitality was measured using the vigor subscale (six items) of the Dutch version of Utrecht Work Engagement Scale (UWES),^18^ adapted to a nonwork context. A subset of five items of the Individual Work Performance Questionnaire (IWPQ)^19^ was used to measure work performance. Presenteeism was assessed using four questions developed to evaluate the effectiveness of the current intervention program.^17^Table 1 provides an overview of these outcome measures, including the items and response options, the reliability at baseline, and the operationalization.

Individual sickness absence data were derived from company records. The percentage sickness absence in a month was based on the number of absence days in a month and the amount of fulltime-equivalent (FTE) a person was working. For the analysis, cumulative sickness absence data over 12 months before T0 and T2 were used (Note that the 12-month period preceding T2 partly overlaps with the intervention period [ie, the last 2 months of the intervention period before T1]). No sickness absence data were collected for T1 due to the short timeframe of five months between T0 and T1. The average percentage of sickness absence for each person was calculated over the available months, including only those participants of whom data on sickness absence were available of at least nine months. Because sickness absence has a skewed distribution with a substantial fraction clustered at the value zero, it was dichotomized into 0 (average percentage of sickness absence = 0) and 1 (average percentage of sickness absence >0). The percentage of respondents with an average percentage of sickness absence more than 0 in the defined time period is reported.

### Statistical Analysis

All participants of whom data were available at the start of the intervention (ie, baseline), whether or not they completed the study, were initially included in the analyses. Next, participants who reported unrealistic data with regard to sitting time were removed from the sample, using a reported sitting time of more than 16 hours and 40 minutes (ie, 1000 minutes) on a regular working day as the cut-off point. This cut-off point is in line with previous work.^20^ In addition, those reporting zero minutes of total sitting time on a working day were also considered to be unrealistic. Of the final sample of participants, all available data were included in the analysis. The distribution of all measurements was considered normal, except for MVPA, for which a square root transformation was taken.

Because longitudinal data collection often has to deal with missing data due to drop-out of participants, demographics, sitting time, and the other primary outcome measures, vitality, work performance, and presenteeism were compared at baseline between participants who completed all measurements (“completers”) and participants who had missing measurements at T1 and/or T2 (“drop-outs”). When no significant differences are found, data can be assumed to be missing at random and will introduce minimal bias to further analyses. Variables were evaluated with independent *t* tests for continuous variables and χ^2^-tests for categorical variables.

To determine change over time for sitting time, vitality, work performance, and presenteeism, linear mixed model analysis was performed, which is permitted as long as missing at random is assumed for incomplete data.^21^ The time variable was used at the first level of the measurement model to investigate whether there was a significant increase or decrease over time (from baseline to T1 and from baseline to T2), with the three time points being T0 (baseline), T1 (after five months), and T2 (after 15 months). All models included a random intercept to allow for variation between participants in baseline score. At the second level of the model, effects of covariates were estimated (gender, age (mean centered at 42.1), level of education (lower vs higher educated), employment status (<36 vs ≥36 hours a week), BMI (<25 vs ≥25 kg/m^2^), and MVPA (minutes/week). In addition, unadjusted analyses (with only the time variable as predictor) were performed and compared with results of the adjusted analyses.

Generalized linear mixed models were used to estimate the relationship between sitting time and vitality, work performance and presenteeism, and possible interaction effects over time. The time variable and sitting time were included at the first level of the model in order to determine main effects. Next, interaction variables were included for sitting time^∗^T1 and sitting time^∗^T2 (ie, the original sitting time variable would provide the interaction value for T0).

To determine change over time for the dichotomous outcome measure sickness absence, logistic regression analysis was used to calculate the odds ratio (OR). Similarly, logistic regression was used to estimate the relationship between sitting time and sickness absence and the possible interaction effect over time. The time variable and sitting time were included at the first level of the model in order to determine main effects. Next, the interaction variables were included for sitting time^∗^T2.

## RESULTS

### Number of Participants

At the start of the intervention, data were available for 433 participants (86% response rate). Because of unrealistic data (ie, reporting zero minutes or more than 1000 minutes of sitting time on a regular working day), 37 participants were removed from further analysis, leaving a final sample of 396 participants with data at T0 (91% of those who responded). Of these respondents, 301 also had data on T1, 172 also had data on T2, and 153 respondents had data on all three measurement points. Statistical comparison showed no significant demographical differences between the final sample and the 37 participants removed from analysis.

### Background Characteristics and Primary Outcome Measures

Table 2 provides an overview of the background characteristics of the sample and the primary outcome measures. Participants were on average in their forties, highly educated, and two-thirds worked full-time. Sitting time on a working day was high (on average 10.5 hours). “Completers” (participants who completed all measurements) were compared with “drop-outs” (participants who had missing measurements at T1 and/or T2) on background characteristics and primary outcome measures (at T0). No significant differences were found, indicating no evidence for selective drop-out.

### Results of Sitting Time and Work-Related Outcomes

The results of sitting time and work-related outcome measures are presented in Table 3, including the results of multilevel regression analyses of each variable. Because no difference was found between unadjusted and adjusted analysis, Table 3 only presents the results of the unadjusted analyses.

Sitting time showed a significant decrease in the short-term, directly after the intervention (T0-T1) as well as in the long-term (T0-T2) of about 25 minutes. Positive outcomes were also observed for work performance, which increased significantly in the short-term (T0-T1) and in the long-term (T0-T2). Sickness absence data could only be compared in the long-term (T0-T2) and the results showed a significant decrease. No significant changes were observed for vitality and presenteeism.

### Association Between Changes in Sitting Time and Work-Related Outcomes

Table 4 summarizes to what extent changes in sitting time are associated with changes in vitality, work performance, presenteeism, and sickness absence. First, the main effects were established between sitting time and the work-related outcomes in general. Second, the interaction variables were included to obtain the relationship between sitting time and the work-related outcomes for each time point separately.

A significant negative main effect was observed between vitality and sitting time: sitting less is associated with higher vitality scores (*b* = −0.0006, *P* *=* 0.000). The significant interaction effect at T0, with nonsignificant interaction effects at T1 and T2, suggests that this effect primarily existed at baseline, before the intervention took place. For work performance, presenteeism, and sickness absence, no main effects were found. However, for presenteeism, a significant negative interaction effect was found at T1 (*b* = −0.0008, *P* = 0.005). This suggests that shortly after the intervention, sitting fewer hours was associated with higher presenteeism scores. This relationship was weaker at T0 and T2, which explains why no significant main effect was found.

## DISCUSSION AND CONCLUSIONS

In the past years, increasing attention in the literature has been paid to the negative health effects of a sedentary lifestyle. Although the evidence is still incomplete and sometimes contradictory, it seems justified from a health perspective to implement interventions aimed at reducing sedentary behavior. In the working population, many job tasks involve sedentary components, and in an increasing number of jobs, workers are more or less forced to sit almost the whole working day without proper breaks. In these jobs, work can be seen as a causal factor of sedentary behavior, which calls for preventive action by employers, given the adverse health effects of sedentary behavior. Thus, employers will have to search for interventions aiming to reduce the sedentary behavior of their workers. Apart from the employees, employers could benefit from these interventions as well when such a decrease results in healthier, vital workers showing better work performance and less sickness absence. However, until now, there are few studies that have explored whether such a relationship between sitting time on a working day and work-related outcomes is indeed substantial and thus relevant for the work setting.

We first explored whether the occupational health promotion program, focusing on increasing self-management to perform healthy behaviors (including sedentary behavior but not in particular), resulted in decreased sitting time on a working day. A significant and sustainable decrease of sitting time on a working day, both immediately after the intervention and at follow-up, of about 25 minutes was found. It can be expected that an intervention specifically targeting sitting time will result in a larger effect, of which evidence was found in two recent systematic reviews.^22,23^ The significant reduction in sitting time found can partly be explained by the fact that sitting time was high at baseline, which provided a lot of room for improvement. Comparable high sitting times are found in other studies using self-report measurement of sitting time during working days.^20,24–26^ However, it is possible that these high self-reports are overestimated: a recent study showed that self-reported total sedentary time overestimated the objectively measured sedentary time in workers who reported high averages of self-reported sedentary time.^27^

Second, we explored to what extent the decrease in sitting time was associated with changes in vitality, work performance, presenteeism, and sickness absence. We did find a significant though marginal association with vitality: workers who were less sedentary felt more vital. This association primarily existed at baseline, before the intervention took place. So, even though sitting time and vitality were associated, changes in sitting time were not directly associated with changes in vitality.

We did not find any main effects for the other work-related outcomes, but we did find a significant negative relationship between sitting time and presenteeism at short-term follow-up. This suggests that participants who reported higher scores on presenteeism directly after the intervention did benefit most in terms of reduced sitting time.

There are few studies available to confirm these findings. In a cohort of 4436 UK office-workers, those reporting average to high work vigor (subscale of the UWES) were less likely to report high occupational sitting times (ie, less likely to sit more than 420 minutes/day).^10^ Besides, a significant correlation was found between higher job performance (using four items on a seven-point Likert scale) with lower occupational sitting times. In another cross-sectional study among 108 Australian office employees, the relationship between employee presenteeism [measured using the Work Limitations Questionnaire (WLQ) Index score] and objectively measured total sedentary time on a working day [OR = 2.15; 95% confidence interval (95% CI): 0.99 to 4.69] approached significance.^11^ The constrained variability among employees in this study, with high levels of sedentary behavior, low levels of presenteeism, and little variability in WLQ Index scores, was mentioned by the authors as a possible explanation of finding no significant association. In the recent cross-sectional study by Guertler et al,^12^ the opposite was found: in 710 Australian workers who completed an online survey, higher work-related sitting was significantly associated with lower presenteeism when controlling for other lifestyle behaviors. When examining this unexpected association separately by occupation, high work-related sitting time was associated with lower presenteeism only in “professionals” (managers and administrators, and [associate] professionals), but not in white-collar and blue-collar workers. In conclusion, available literature supports our findings of significant but marginal associations between sitting time and important work-related outcomes. High-quality studies are needed to explore these associations in more detail and to investigate whether there is a causal relationship between changes in sitting time and changes in work-related outcomes.

Our study is one of the first studies to focus on sitting time and work-related outcomes in a longitudinal design, including long-term measurement. Measurement of sitting time was relatively comprehensive, involving questions specifically aimed at sedentary activities on a working day. In addition, we had access to the registered company sickness absence data. However, a severe limitation of our study was the absence of a control group and randomization. This is a major drawback in research involving worker populations: companies are seldom willing to allow a randomized controlled design, because they want all employees to benefit from the program at the same time. As a result of using this study design, no statements can be made on the effectiveness of the intervention program on sitting time and the work-related outcomes. In addition, drop-out rates were substantial, which is a common problem in intervention studies.^28^ However, there was no indication of selection and still a substantial number of workers participated in the long-term follow-up measurements. Furthermore, we analyzed the data collected by the company that delivered the health promotion program. They used a comprehensive questionnaire developed specifically to measure the effects of their program on work-related outcomes, with many questions derived from various validated questionnaires measuring specific outcomes. However, the sets of questions we used in our current analyses were as such not validated, which could have influenced the results. Finally, as a result of unrealistic high sitting time data of some participants, we decided to use a maximum of 1000 minutes a day as a cut-off point, which is in line with Kazi et al.^20^ This cut-off point is slightly more conservative than the one used by Bennie et al,^29^ who truncated their sitting time data at 960 minutes (16 hours) under the assumption that an otherwise healthy ambulatory adult would be mobile for at least 8 hours each day (eg, light intensity walking from place to place, around the house, at work, etc.).

Despite these limitations, we conclude that a decrease in sitting time has significant though marginal associations with some relevant work-related outcomes. As it is known that these outcomes are related to many other factors that are far more prominent at the workplace, such as physical and psychosocial working conditions and health,^30^ the fact that we did see some significant associations is as such a relevant finding. It justifies further research on the impact of interventions to decrease sitting time on major work-related outcomes as vitality, work performance, presenteeism, and sickness absence.

## Figures and Tables

**TABLE 1:** Overview of Outcome Measures, Items and Response Options, Reliability at Baseline (T0), and Operationalization

Outcome Measure	Items	Response Options	Reliability at Baseline (T0)	Operationalization
Sitting time	On average, how many hours and minutes do you sit on a regular working day (eg, time behind a desk, watching a screen, reading a book, etc.)?	Number of hours and minutes per day	n.a.	Sum of 1, 2, 3, and 4 in minutes per working day
	(1) during work			
	(2) during work breaks			
	(3) during travelling to/from work			
	(4) during leisure time			
Vitality^*^	(1) I feel bursting with energy.	Scale 0 (never) to 6 (always)	α = 0.85	Mean of 6 items
	(2) I feel strong and vigorous.			
	(3) When I get up in the morning, I feel like getting started.			
	(4) When I am busy, I can continue for a very long time.			
	(5) I am very resilient, mentally.			
	(6) I always persevere, even when things do not go well.			
Work performance^†^	(1) For the past three months, I was able to focus on the result of my work.	Scale 1 (seldom or never) to 5 (always)	α = 0.71	Mean of 5 items
	(2) For the past three months, I was able to plan my work to make sure it was finished on time.			
	(3) For the past three months, I was able to do my work with the least time and effort.			
	(4) For the past three months, I put effort in developing my job skills.			
	(5) For the past three months, I came up with creative solutions to new problems.			
Presenteeism	(1) How often are you at work while unable to perform at an optimal level?	Scale 1 (every day) to 7 (never)	α = 0.72	Mean of 4 items
	(2) How often are you at work while actually not feeling well/feeling sick?			
	(3) How often are you at work while mentally being somewhere else?			
	(4) How often do you perform less than you are able to?			

α, Cronbach alpha; n.a., not available.

^*^Vitality items of the Utrecht Work Engagement Scale (UWES)^18^ were adapted to a nonwork context.

^†^A subset of items of the Individual Work Performance Questionnaire (IWPQ)^19^ was used.

**TABLE 2:** Background Characteristics and Primary Outcome Measures^∗^ of All Participants at Baseline (T0), Completers and Drop-Outs (Participants who Missed at Least One Measurement) in Means (SD) or %

	All Participants at Baseline (*n* = 396)	Completers (*n* = 153)	Drop-Outs *(*n = 243)	*P*
Age, yrs [mean (SD)]	42.1 (8.6)	42.1 (8.3)	42.0 (8.8)	0.91
Gender (% women)	52.8	50.3	54.3	0.44
Education (% highly-educated)^†^	63.4	66.0	61.7	0.39
Employment status (% ≥36 hrs per week)	65.9	67.3	65.0	0.64
BMI (% ≥25)	47.2	47.1	47.5	0.93
MVPA [min/week, mean (SD)]	149 (154)	152 (153)	146 (155)	0.71
Sitting time [min/working day, mean (SD)]	630 (127)	629 (132)	631 (123)	0.92
Vitality [scale 0–6, mean (SD)]	3.86 (.73)	3.88 (.73)	3.85 (.73)	0.71
Work performance [scale 1–5, mean (SD)]	3.59 (.63)	3.65 (.65)	3.55 (.61)	0.11
Presenteeism [scale 1–7, mean (SD)]	5.94 (.65)	5.95 (.60)	5.93 (.68)	0.71
Sickness absence (% absent)^‡^	47.2	45.5	47.8	0.66

BMI, body mass index (kg/m^2^); MVPA, moderate to vigorous physical activity; SD, standard deviation.

^*^Higher values indicate better results, except for sitting time and sickness absence; completers, participants who completed all measurements; drop-outs, participants who had missing measurements at T1 and/or T2.

^†^Highly educated: bachelor or master degree.

^‡^Sickness absence: percentage of participants who were one or more days absent due to sickness in the previous 12 months (only participants with absence data of at least nine months were included in the analyses; all participants at baseline *n* = 360; completers *n* = 142; drop-outs *n* = 218).

**TABLE 3:** Number of Participants, Means (SD), or % of Outcome Measures^∗^ at Baseline (T0), 5 Months (T1), and 15 Months (T2), and the Results of (Unadjusted) Multilevel Regression Analyses and Logistic Regression

	T0 (*n* = 396)		T1 (*n* = 301)		T2 (*n* = 172)		T0-T1		T0-T2	
Continuous Outcomes	Mean	SD	Mean	SD	Mean	SD	*b* (95% CI)	*P*	*b* (95% CI)	*P*
Sitting time	630	127	604	123	605	132	−25.12	**0.001**	−23.53	**0.011**
(minutes/ working day)							(−39.70 to −10.54)		(−41.56 to −5.50)	
Vitality (scale 0–6)	3.86	0.73	3.86	0.71	3.94	0.76	0.02 (−0.05 to 0.08)	0.608	0.07 (−0.01 to 0.15)	0.073
Work performance (scale 1–5)	3.59	0.63	3.72	0.60	3.73	0.57	0.11 (0.04 to 0.18)	**0.001**	0.14 (0.06 to 0.22)	**0.001**
Presenteeism (scale 1–7)	5.94	0.65	5.95	0.64	5.98	0.67	0.01 (−0.05 to 0.08)	0.678	0.02 (−0.06 to 0.11)	0.560

Categorical Outcome	T0 (*n* = 360)	T2 (*n* = 131)	T0-T1		T0-T2	
	%	%	OR	*P*	OR	*P*
Sickness absence^†^ (% absent)	47.2	34.4	n.a.	n.a.	0.52	**0.00**

Bold: significant change (*P* < 0.05). CI, confidence interval, n.a., not available; SD, standard deviation.

^*^Higher values indicate better results, except for sitting time and sickness absence.

^†^Only participants with absence data of at least nine months were included in the analyses.

**TABLE 4:** Main Effects Between Sitting Time and Work-Related Outcomes, and the Interaction Effects Between Sitting Time and Time (ie, Baseline [T0], 5 Months [T1], and 15 Months [T2])

	Main Effect		Interaction T0		Interaction T1		Interaction T2	
Continuous Outcomes	*b* (95% CI)	*P*	*b* (95% CI)	*P*	*b* (95% CI)	*P*	*b* (95% CI)	*P*
Vitality	−0.0006	**0.000**	−0.0008	**0.001**	0.0003	0.240	0.0003	0.422
	(−0.0009 to −0.0002)		(−0.0011 to −0.0003)		(−0.0002 to 0.0008)		(−0.0004 to 0.0009)	
Work performance	−0.0002	0.145	−0.0001	0.777	−0.0004	0.133	−0.0001	0.671
	(−0.0005 to 0.0001)		(−0.0005 to 0.0004)		(−0.0010 to 0.0001)		(−0.0008 to 0.0005)	
Presenteeism	−0.0002	0.189	0.0001	0.524	−0.0008	**0.005**	−0.0004	0.241
	(−0.0006 to 0.0001)		(−0.0003 to 0.0006)		(−0.0014 to −0.0003)		(−0.0011 to 0.0003)	

	Main Effect		Interaction T0		Interaction T1		Interaction T2	
Categorical Outcome	*b* (SD)	*P*	*b* (SD)	*P*	*b* (SD)	*P*	*b* (SD)	*P*
Sickness absence	−0.001	0.378	0.000	0.644	n.a.	n.a.	0.001	0.594
	(0.001)		(0.001)				(0.002)	

Bold, significant association (*P* < 0.05). CI, confidence interval; n.a., not available.
